# Interactions between *Bifidobacterium* and *Bacteroides* and human milk oligosaccharides and their associations with infant cognition

**DOI:** 10.3389/fnut.2023.1216327

**Published:** 2023-06-29

**Authors:** Seoyoon Cho, Tinu M. Samuel, Tengfei Li, Brittany R. Howell, Kristine Baluyot, Heather C. Hazlett, Jed T. Elison, Hongtu Zhu, Jonas Hauser, Norbert Sprenger, Weili Lin

**Affiliations:** ^1^Department of Biostatistics, University of North Carolina at Chapel Hill, Chapel Hill, NC, United States; ^2^Nestle Product Technology Center-Nutrition, Société des Produits Nestlé S.A., Vevey, Switzerland; ^3^Biomedical Research Imaging Center, University of North Carolina at Chapel Hill, Chapel Hill, NC, United States; ^4^Department of Radiology, University of North Carolina at Chapel Hill, Chapel Hill, NC, United States; ^5^Fralin Biomedical Research Institute at VTC, Department of Human Development and Family Science, Virginia Polytechnic Institute and State University, Roanoke, VA, United States; ^6^Department of Psychiatry, University of North Carolina at Chapel Hill, Chapel Hill, NC, United States; ^7^Institute of Child Development, University of Minnesota, Minneapolis, MN, United States; ^8^Nestlé Institute of Health Sciences, Société des Produits Nestlé S.A., Lausanne, Switzerland

**Keywords:** human milk, infant gut microbiota, early cognitive development, Mullen Scales of Early Learning, human milk oligosaccharides, random linear mixed effects model, group lasso

## Abstract

While ample research on independent associations between infant cognition and gut microbiota composition and human milk (HM) oligosaccharides (HMOs) has been reported, studies on how the interactions between gut microbiota and HMOs may yield associations with cognitive development in infancy are lacking. We aimed to determine how HMOs and species of *Bacteroides* and *Bifidobacterium* genera interact with each other and their associations with cognitive development in typically developing infants. A total of 105 mother-infant dyads were included in this study. The enrolled infants [2.9–12 months old (8.09 ± 2.48)] were at least predominantly breastfed at 4 months old. A total of 170 HM samples from the mothers and fecal samples of the children were collected longitudinally. Using the Mullen Scales of Early Learning to assess cognition and the scores as the outcomes, linear mixed effects models including both the levels of eight HMOs and relative abundance of *Bacteroides* and *Bifidobacterium* species as main associations and their interactions were employed with adjusting covariates; infant sex, delivery mode, maternal education, site, and batch effects of HMOs. Additionally, regression models stratifying infants based on the A-tetrasaccharide (A-tetra) status of the HM they received were also employed to determine if the associations depend on the A-tetra status. With *Bacteroides* species, we observed significant associations with motor functions, while *Bif. catenulatum* showed a negative association with visual reception in the detectable A-tetra group both as main effect (value of *p* = 0.012) and in interaction with LNFP-I (value of *p* = 0.007). Additionally, 3-FL showed a positive association with gross motor (*p* = 0.027) and visual reception (*p* = 0.041). Furthermore, significant associations were observed with the interaction terms mainly in the undetectable A-tetra group. Specifically, we observed negative associations for *Bifidobacterium* species and LNT [*breve* (*p* = 0.011) and *longum* (*p* = 0.022)], and positive associations for expressive language with 3′-SL and *Bif. bifidum* (*p* = 0.01), 6′-SL and *B. fragilis* (*p* = 0.019), and LNFP-I and *Bif. kashiwanohense* (*p* = 0.048), respectively. Our findings suggest that gut microbiota and HMOs are both independently and interactively associated with early cognitive development. In particular, the diverse interactions between HMOs and *Bacteroides* and *Bifidobacterium* species reveal different candidate pathways through which HMOs, *Bifidobacterium* and *Bacteroides* species potentially interact to impact cognitive development in infancy.

## 1. Introduction

Over the last few decades, there has been an increase in studies examining the effects of the gut microbiome on the health of the host (1–23). Numerous studies in both animals and humans have identified connections between the gut microbiome and various health aspects (10). These include cognition (23), obesity, cardiovascular diseases, intestinal conditions, immunity (1, 4, 5, 15), as well as mental health such as depression, anxiety (3, 7, 8, 12, 14, 16, 18, 19), and emotional behaviors (9). A lack of exposure to diverse microorganisms could lead to an increased risk of allergic diseases (15). Rogers et al. investigated the bidirectional relationship between gut microbes and the central nervous system. They found that treating mother-deprived rats with *Bifidobacterium infantis* normalized their immune response, and that exposing mice with gastrointestinal inflammation and infection to *Bifidobacterium longum* normalized their anxious behavior (6). Carlson et al. (22) reported that the *Bacteroides*-abundant cluster had improved early learning composite score and language related scales. Finally, on the species level, Savignac et al. (3) showed that the mice fed with *Bifidobacterium longum* or *Bifidobacterium breve* exhibited improved cognition when compared to the controls.

Although the aforementioned results have demonstrated the interplay between the gut microbiome and the host’s health, Zafar and Saier pointed out the potential beneficial effects of *Bacteroides* when metabolizing polysaccharides and oligosaccharides. *Bacteroides* serves as an immunomodulator, and provides nutrients and vitamin K to both the host and other intestinal microbial residents (1). In addition, among the species in the *Bacteroides* genus, the *B. fragilis* and *B. vulgatus* species are also known to metabolize human milk oligosaccharides (HMOs) (24–26). Many studies have also examined the associations between HMOs and *Bifidobacterium* species, which are well-known consumers of HMOs as prebiotics. For example, Matsuki et al. (27) showed that some *Bifidobacterium breve* strains use fucosyllactose in breast-fed infants, leading to higher *Bifidobacterium* abundance and metabolic signatures characteristic of higher *Bifidobacterium* metabolic activity. Collectively, these findings strongly support the potential interactions between specific gut microbes and HMOs, providing insight into how different HMOs may alter infant gut microbiota composition and function (2, 28–32).

Human milk oligosaccharides, on the other hand, have also been independently studied regarding their potential health benefits, including cognition during infancy. Specifically, 3′-(3′-SL) and 6′-sialyllactose (6′-SL), and 2′-fucosyllactose (2′-FL), were shown to improve and/or be associated with general cognitive ability (33–37), motor skills (36), learning (36, 38–42), language (21), spatial cognition ability (43), and anxiety reduction (44). Jorgensen et al. reported that infants receiving human milk (HM) high in either sialylated or fucosylated HMOs exhibited increase in language abilities at 18 months (45). Cho et al. further extended their findings and showed that 3′-SL had positive associations with language abilities in breast-fed infants who received HM containing detectable A-tetrasaccharide (A-tetra) during infancy (21).

Given these findings, it is highly plausible that a triad relation exists among gut microbiota, HMOs, and cognition during early infancy. That is, both gut microbiota and HMOs could independently and/or through the interactions of the two yield associations with cognitive development in infants. To this end, we aimed to discern if the potential interactions between HMOs and specific gut microbiota species in the *Bifidobacterium* and *Bacteroides* genera, are associated with cognition assessed using Mullen Scales of Early Learning (MSEL) (46) during infancy. Specifically, we hypothesized that while HMOs and species of *Bifidobacterium* and *Bacteroides* may be independently associated with cognition, the interactions between them could also be associated with different aspects of cognitive development during the first year of life. Additionally, recent reports suggested that the presence/absence of A-tetra in HM depends on the secretor status and blood group (A or AB) (47–49), which could potentially influence the results of this triad association. In a study by Cho et al., it was found that the association between HMOs and cognition depended on the presence or absence of A-tetra in HM (21). Therefore, we further hypothesized that the associations with cognition with the identified interactions between HMOs and gut microbiota may also depend on the presence or absence of A-tetra in the HM that infants received.

## 2. Materials and methods

### 2.1. Study subjects

Parents enrolled in this study provided written informed consent for the participation of both themselves and their infants. The University of North Carolina at Chapel Hill and University of Minnesota Institutional Review Boards approved all study activities. Using site-based research registries, subjects were enrolled from both universities. Local newborn nurseries, institutional centers with research interest on early brain development, local flyers, and university listservs were additionally used for recruitment. The inclusion criteria were: (1) birth at 37–42 weeks of gestational age; (2) appropriate birth weight for gestational age; and (3) no major pregnancy and delivery complications. The exclusion criteria included: (1) adopted child; (2) presence of autism, intellectual disability, schizophrenia, or bipolar disorder related first degree; (3) less than 2 kg of birth weight; (4) neonatal hypoxia (10 min APGAR <5); (5) having illness requiring more than 2 days of newborn intensive care unit stay; (6) chromosomal or major congenital abnormality; (7) abnormal magnetic resonance in previous MRI; (8) significant developmental delay or medical illness, or significant genetic or medical conditions impacting growth, development, or cognition (including visual/hearing impairment); (9) contraindication in MRI; and (10) maternal pre-eclampsia, HIV status, placental abruption, and alcohol or illicit drug use during pregnancy. Finally, additional inclusion criteria for subjects included in this study were infants younger than 12 months old and exclusively/predominantly breastfed during the first 4 months of life, defined as the infants who were fed less than 20 g or four teaspoons per day of complementary foods/liquids (water, apple juice, etc.), and non-formula.

### 2.2. Human milk collection and analyses

Human milk samples were obtained from the right breast using a hospital-grade, electric Medela Symphony breast pump at each visit. To ensure that the collected HM samples represented HM composition at each feeding, the samples were gathered until no more HM was expressed. Additionally, whenever possible, HM samples were standardized to the second feed of the day so that the diurnal variation of HM compositions could be minimized. The weight and volume of the samples were recorded and then vortexed at the highest speed for 2 min. A graduated cylinder was used for volume measurement with extra care to avoid bubbles. The total fat content was then measured using mid-infrared spectroscopic analyses (MIRIS Human Milk Analyzer) to ensure that the total fat content, which indicates the quality of milk sampling, was within the expected range of 2.5 mL. Lastly, from the collection bottle, an aliquot of the minimum 30 mL of volume was transferred to a 50 mL polypropylene Falcon tube. Repeat pipette and appropriate tips were used to make 11 aliquots of 1 mL in 1 mL Eppendorf tubes, and nine aliquots of 2 mL in 2 mL Eppendorf tubes for storage in a − 80°C freezer after the collection was done.

For HMO quantifications, a representative 1 mL aliquot of HM was shipped to Neotron Spa (Italy) on dry ice. HMO analyses were done following Austin and Benet (50). Analyses were done using liquid chromatography with fluorescence detection after labeling with 2-aminobenzamide. HMOs were quantified using a standard ultra-high liquid chromatography (UHPLC) system. The system was equipped with a fluorescence detector, which is a two-way 10 port high pressure switching valve and two columns, a VanGuard BEH amide (1.7 μm, 2.1 mm × 50 mm; Waters Corp., Milford, United States) and an Acquity BEH Glycan (1.7 μm, 2.1 mm × 150 mm; Waters Corp., Milford, United States). Neotron used the method developed by the Nestlé Research team, which is ISO17025 certified and a reference human milk sample was included in their analyses to ensure the correct performance of the method. Using standard curves with authentic high purity HMO standards, the following eight HMOs were quantified: 2′-FL, 3-FL, 3′-SL, 6′-SL, Lacto-N-tetraose (LNT), Lacto-N-neotetraose (LNnT), Lacto-N-fucopentaose-I (LNFP-I), and A-tetra (Elicityl SA., Crolles, France). Each analyzed batch of samples were quality controlled. After every 20 samples, quality control samples were included to verify the method performance by allowing only the deviations within ±15% from the expected amounts.

### 2.3. Infant gut microbiota composition and analyses

Stool samples were collected from children’s diapers using the Omnigene Gut sample collection kit (DNA GenoTek, Ontario, Canada) 24 h before, during, or after in-person visits. The collected samples should be stable for up to 60 days in a collection tube and were processed within a week using the following steps. Fecal samples were loosened by placing them in a dry bead bath. A sterile transfer pipette was used to transfer a fecal sample into Eppendorf tubes. The fecal sample was then evenly split between two 1.5 mL Eppendorf tubes, frozen immediately, and stored in a − 80°C freezer. Finally, all collected fecal samples were shipped to CosmosID Inc. (Germantown, MD, United States) for further analyses as detailed below.

### 2.4. DNA extraction, library preparation, and sequencing

Following the manufacturer’s protocol, using the QIAGEN DNeasy PowerSoil Pro Kit (Qiagen, Germantown, MD, United States), DNA from samples was isolated. Quantification of the extracted DNA samples was done using Qubit 4 fluorometer and Qubit™ dsDNA HS Assay Kit (Thermofisher Scientific, MA, United States).

Preparation of DNA libraries was done using the Nextera XT DNA Library Preparation Kit (Illumina, San Diego, CA, United States) and IDT Unique Dual Indexes with total DNA input of 1 ng. Using a proportional amount of Illumina Nextera XT fragmentation enzyme, genomic DNA was fragmented. To each sample, unique dual indexes were added and then libraries were constructed after 12 cycles of PCR. With AMpure magnetic Beads (Beckman Coulter, Brea, CA, United States), DNA libraries were purified and eluted in QIAGEN EB buffer. Libraries were then sequenced on an Illumina NovaSeq 6000 System with S4 Flow Cell.

### 2.5. Bioinformatics analysis

Unassembled sequencing reads were directly analyzed using CosmosID-HUB Microbiome Platform (CosmosID Inc., Germantown, MD, United States). In short, the platform employed curated genome databases together with a high-performance data-mining algorithm allowing rapid disambiguation of hundreds of millions of metagenomic sequence reads into the discrete microorganisms engendering the particular sequences.

Raw data were backed up to Amazon AWS and run through fastqc for quality checks upon data generation. A multiqc report was generated to ensure the conformation of read depth thresholds, and to check that there was no abnormality with read quality, duplication rates, or adapter content. Taxonomic results were checked on the http://app.cosmosid.com platform to ensure no contamination nor barcoding issues. For statistical significance of the results, the filtering threshold was based on statistical scores determined by analyzation of a large number of diverse metagenomes.

Relative abundance of species of *Bifidobacterium* and *Bacteroides* was employed for our analyses. To avoid biases from outliers, the double median absolute deviation approach (51) was employed to remove outliers. Subsequently, relative abundance of each species was summed over all the samples from all the subjects and only the species with a summation greater than one were used in our analyses since including species with infinitesimal abundance does not convey much information and could hamper the efficiency of the analysis with an unnecessarily larger number of variables.

### 2.6. Mullen Scales of Early Learning

The MSEL, a validated and widely used infant cognitive development assessment tool, comprises of five subdomains: fine motor, gross motor, visual reception, receptive language, and expressive language (46). An early learning composite score which is consistent with the Developmental Quotient score for infants was derived using all subdomain scores excluding gross motor. Trained staff administered the MSEL assessment at every visit.

### 2.7. Statistical modeling and analyses

The R version 4.0.3 (The R Foundation for Statistical Computing, Vienna, Austria) was used for all statistical analyses. Relative abundances of the selected species were used based on the aforementioned criteria. Interaction terms were included to capture the dependence between infant gut microbiota and HMOs. The relative abundances and the HMO concentrations were first standardized to ensure fair comparisons among all gut microbiota species and HMOs prior to subsequent analyses. We fitted the following models to examine the relationship between cognition, HMOs and gut microbiota: an unstratified model and two stratified models based on A-tetra status in HM. The unstratified model used the eight HMO concentrations from all study subjects. Thus, the estimated effects were associations between HMOs and infant cognition. In contrast, the stratified models only used the HMO concentrations from a subgroup of the subjects. Thus, the estimated effects captured the associations between infant cognition and HMOs from homogeneous subjects depending on the A-tetra status. For example, in the A-tetra+ stratified model, the HMO concentrations were used for association analyses for infants whose mothers produced HM with detectable A-tetra, but not for infants whose mothers did not. Microbiota relative abundances were used for all infants, but interaction terms between HMO and microbiota species were only included for those fed with HM with detectable A-tetra. Similarly, the A-tetra-stratified model included HMOs from only the infants with mothers with undetectable A-tetra HM. Collectively, if consistent significant associations were observed for all three models, it implied that the associations were independent of the A-tetra status or otherwise the associations depended on the A-tetra status.

All models were adjusted for infant sex, delivery mode, maternal education, site, and batch of HMO analyses.

Unstratified

$$\begin{array}{l} Mullen=\beta_{0}+\beta_{1}adjustingcovariates\\ \;\;\;\;\;\;\;\;\;\;\;\;\;\;\;\;\;\;\;\;\;\;+\beta_{2}HMO+\beta_{3}MB+\gamma_{1}HMO\times MB+\epsilon \end{array}$$

Stratified based on A-tetra+

$$\begin{array}{l} Mullen=\beta_{0}+\beta_{1}adjustingcovariates\\ \;\;\;\;\;\;\;\;\;\;\;\;\;\;\;\;\;\;\;\;\;\;\;+\beta_{2}HMO\left(Atetra+\right)+\beta_{3}MB\\ \;\;\;\;\;\;\;\;\;\;\;\;\;\;\;\;\;\;\;\;\;\;\;+\gamma_{1}HMO\left(Atetra+\right)\times MB\\ \;\;\;\;\;\;\;\;\;\;\;\;\;\;\;\;\;\;\;\;\;\;\;\;+\beta_{4}I\left(Atetra-\right)+\epsilon . \end{array}$$

Stratified based on A-tetra-

$$\begin{array}{l} Mullen=\beta_{0}+\beta_{1}adjustingcovariates\\ \;\;\;\;\;\;\;\;\;\;\;\;\;\;\;\;\;\;\;\;\;\;\;+\beta_{2}HMO\left(Atetra-\right)+\beta_{3}MB\\ \;\;\;\;\;\;\;\;\;\;\;\;\;\;\;\;\;\;\;\;\;\;+\gamma_{1}HMO\left(Atetra-\right)\times MB\\ \;\;\;\;\;\;\;\;\;\;\;\;\;\;\;\;\;\;\;\;\;\;+\beta_{4}I\left(Atetra+\right)+\epsilon \end{array}$$

Here, *HMO* and *MB* correspond to the eight HMO quantifications and the relative abundances of the species from *Bifidobacterium* and *Bacteroides* genera, respectively. In the stratified models, *HMO(Atetra+)* and *HMO(Atetra−)* indicate HMO as a function of A-tetra status, where *HMO(Atetra+)* represents the HMO quantifications from the HM with detectable A-tetra (> the limit of detection of 4.4 mg/L), and vice versa. Furthermore, a binary indicator representing if the HM samples contained detectable [*I(Atetra+)*] or undetectable [*I(Atetra−)*] A-tetra was included for the stratified models and *I(Atetra+)* = 1 when the HM samples were used for A-tetra+ subjects, and 0 for the A-tetra-subjects and vice versa for *I(Atetra−)*.

### 2.8. Variable selection via group LASSO

Finally, to determine how HMOs and gut microbiota and their interactions may be associated with cognition, a two-step approach was employed, which included variable selection and regression analyses. Specifically, the group least absolute shrinkage and selection operator (LASSO) (52) was first used for variable selection of HMOs, microbiota species and their interactions for subsequent regression analyses. The widely used LASSO uses a penalty parameter for each variable to select variables and attains model parsimony (53). While the group LASSO also utilizes the same concept, one fundamental difference is that the penalties are given to a group of variables instead of each variable (52). That is, variables are either selected as a group or not. By applying group LASSO to the interaction models, the corresponding main associations were always chosen when an interaction term was selected so that the model could be interpreted. This step was needed to reduce dimensionality of the included variables and minimize overfitting. The penalty parameter, which influences the selection of the variables, was chosen using 10-fold cross-validation so that it has the best prediction error (54). Since randomness was introduced from the cross-validation, 200 repetitions were done in our study to ensure that the observed results were stable. If the final selected model did not include any interaction terms, the main associations with all eight HMOs and gut microbiota relative abundances were used for subsequent regression analyses. The adjusting covariates were always included in the linear mixed effects model so that these factors could be controlled in the analyses. Finally, linear mixed effects models were fitted with the chosen variables to account for the longitudinal data (55, 56). The dependence within a subject was captured with a random intercept for each infant.

## 3. Results

### 3.1. Subject demographics and descriptive data

A total of 105 mother-infant dyads were included in this study. The infants aged between 2.9 and 12 months old (mean and standard deviation of age: 8.09 ± 2.48 months). Of the 105 mother-infant dyads, 60 visited once while the remaining subjects had at most four visits, leading to a total of 170 MSEL assessments and fecal samples from the infants and 170 HM samples from the mothers. The demographic information of the study subjects and the MSEL are provided in Tables 1, 2, respectively. Except for the expressive language score (*p* = 0.03, A-tetra+: 51.82, A-tetra-: 54.83 mean scores), the MSEL scores were statistically similar between the A-tetra+ and A-tetra-groups.

**Table 1:** Demographic information of the participants.^1^

		Total	A-tetra+	A-tetra−	*p* value^2^
Subjects		105 subjects (170 samples)	36 subjects (61 samples)	69 subjects (109 samples)	
Sex (male)		40 (38%)	15 (42%)	25 (36%)	0.59
Age (months)		8.08 (2.48)	8.13 (2.67)	8.06 (2.38)	0.86^3^
Birth weights (kg)		3.57 (0.45)	3.61 (0.46)	3.55 (0.44)	0.54
Birth lengths (cm)		51.97 (2.54)	52.15 (2.62)	51.87 (2.51)	0.62
Gestation age (months)		9.30 (0.26)	9.30 (0.23)	9.30 (0.30)	0.98
Vaginal birth		83 (79%)	27 (75%)	56 (81%)	0.48
Household Income (*n*)	< 50 k	5	2	3	0.24
	50–75 k	21	6	15	
	75–100 k	18	3	15	
	100–150 k	32	13	19	
	150–200 k	16	6	10	
	200 k <	11	4	7	
Mother education (at least some graduate level)		64 (61%)	21 (58%)	43 (62%)	0.70

1 Means or counts and standard deviations in parentheses.

2 *p* values from the *t*-test or Chi-square test of independence (household income) for comparing between detectable (A-tetra+) and undetectable (A-tetra−) A-tetrasaccharide groups.

3 *p* value calculated as treating each sample to be independent.

**Table 2:** The Mullen Scales of Early Learning scores of the participants.^1^

Mullen Scales of Early Learning	Total (*n* = 170)	A-tetra+ (*n* = 61)	A-tetra− (*n* = 109)	*p* value^2^^,^^3^
Composite score	106.18 (12.48)	104.87 (11.62)	106.91 (12.93)	0.29
Gross motor	50.61 (8.77)	50.75 (8.95)	50.53 (8.70)	0.88
Visual reception	54.24 (9.87)	53.38 (9.21)	54.72 (10.23)	0.38
Fine motor	53.40 (11.43)	52.98 (10.81)	53.63 (11.80)	0.72
Receptive language	50.82 (9.48)	51.39 (8.99)	50.50 (9.78)	0.55
Expressive language	53.75 (8.67)	51.82 (8.03)	54.83 (8.86)	0.03^4^

1 Means and standard deviations in parentheses.

2 *p* values from the *t*-test comparing between detectable (A-tetra+) and undetectable (A-tetra−) A-tetrasaccharide groups.

3 Value of *p* calculated as treating each score to be independent for each visit.

4 Expressive language score shows significant difference between A-tetra+ and A-tetra− subjects.

**Figure 1:**
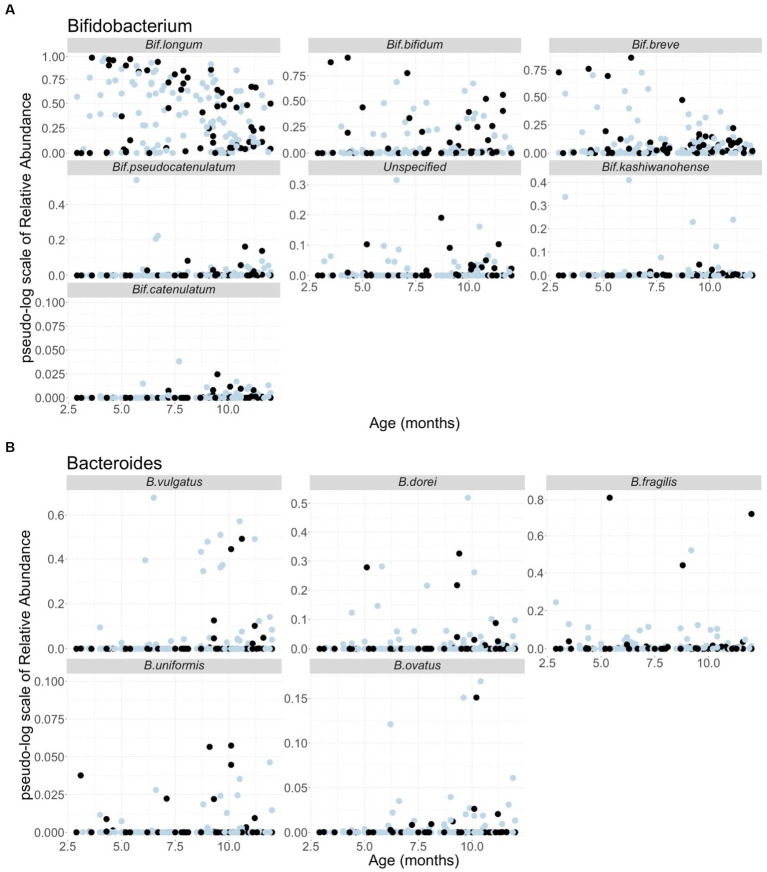
Scatter plots of the pseudo-log scale of the relative abundance of the species used in the analyses, including **(A)** seven species from *Bifidobacterium* genus and **(B)** five species from *Bacteroides* genus. The samples in the detectable A-tetrasaccharide (A-tetra) group are colored in black and the ones in the undetectable A-tetra group are colored in light blue. Age in months is indicated in the *x*-axis.

**Figure 2:**
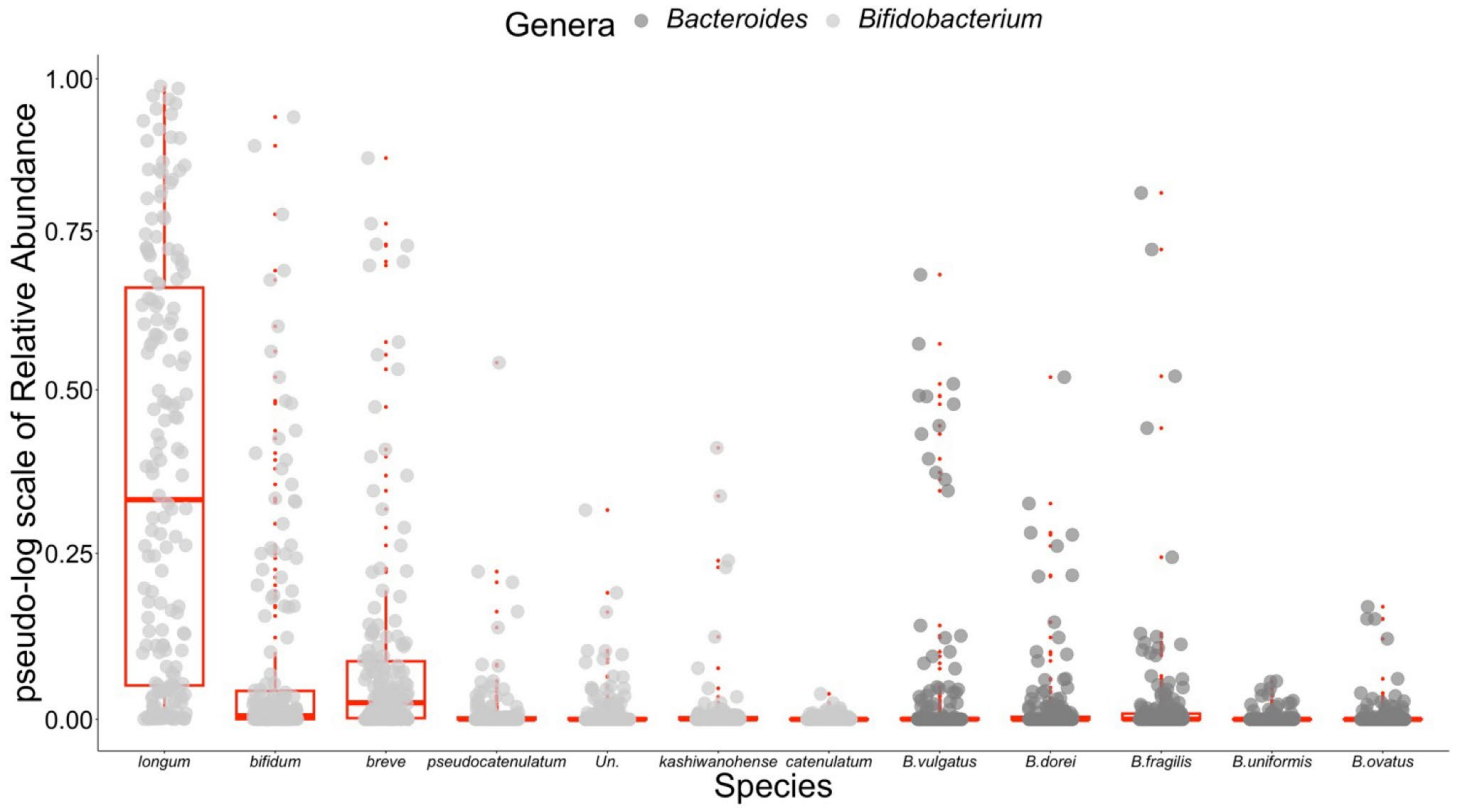
Strip chart for relative abundances of the 12 species used in the analyses. *Bifidobacterium* species (light gray) and *Bacteroides* species (dark gray) in different colors. Boxplot with whiskers added in red for each species. For *Bifidobacterium* species, only the species name included due to visibility. *Un.* for unspecified *Bifidobacterium* species.

Since *Bifidobacterium* and *Bacteroides* are the two most abundant genera in the infant gut microbiota and their potential interaction with HMOs has been widely reported (1, 2, 24–32, 57–62), our analyses focused on these two genera. A total of 37 and 59 species including unspecified species were obtained from the *Bifidobacterium* and *Bacteroides* genera, respectively. As indicated in the Methods section, only the species with a summation of relative abundance over all samples greater than one were used in our analyses, yielding a total of 12 species, including five *Bacteroides* and seven *Bifidobacterium* species. We used *B.* and *Bif.* to represent *Bacteroides* and *Bifidobacterium* when species were indicated hereafter, respectively. Figure 1 shows the temporal characteristics of the pseudo-log-scaled relative abundance of the 12 species and results of the remaining species are provided in Supplementary Figure 1. The black and light blue circles represent A-tetra+ and A-tetra-HM samples, respectively. Evidently, *Bif. longum*, *Bif. bifidum*, and *Bif. breve* exhibited a markedly higher relative abundance than other species in genus *Bifidobacterium* whereas *B. vulgatus*, *B. dorei*, and *B. fragilis* were higher than the remaining two species in genus *Bacteroides* (Figure 2; Table 3). The strip chart for all the 96 species in *Bifidobacterium* and *Bacteroides* genera is provided in Supplementary Figure 2. The relative abundances of *Bif. longum* and *Bif. breve* were negatively associated with age [effect size (ES) = −0.91 and − 0.41 with *p* = 0.004 and 0.012, respectively] while *Bif. catenulatum* and *B. vulgatus* were positively associated with age (ES = 0.01 and 0.26 with *p* = 0.023 and 0.035, respectively). We further evaluated if a nonlinear relationship between relative abundance and age was present by including an additional quadratic age term in the analyses and none exhibited such relationship. Finally, no difference was observed for the standardized relative abundance among the 12 included species between A-tetra+ and A-tetra-HM samples (Table 3).

**Table 3:** The relative abundance of the species from *Bifidobacterium* and *Bacteroides* genera of the participants.^1^

Generum	Species	Total (*n* = 170)	A-tetra+ (*n* = 61)	A-tetra− (*n* = 109)	*p* value^2^^,^^3^
*Bifidobacterium*	*Bif. longum*	0.38 (0.32)	0.38 (0.35)	0.38 (0.30)	0.97
	*Bif. bifidum*	0.09 (0.18)	0.12 (0.22)	0.07 (0.15)	0.17
	*Bif. breve*	0.09 (0.17)	0.10 (0.19)	0.08 (0.15)	0.50
	*Bif. pseudocatenulatum*	0.01 (0.05)	0.01 (0.03)	0.01 (0.06)	0.49
	*Bif. kashiwanohense*	0.01 (0.05)	0.002 (0.01)	0.02 (0.06)	0.03^4^
	*Bif. catenulatum*	0.001 (0.004)	0.001 (0.004)	0.002 (0.005)	0.77
	Unspecified	0.01 (0.04)	0.01 (0.03)	0.01 (0.04)	0.95
*Bacteroides*	*B. vulgatus*	0.04 (0.12)	0.02 (0.09)	0.05 (0.14)	0.08
	*B. dorei*	0.02 (0.07)	0.02 (0.06)	0.02 (0.07)	0.63
	*B. fragilis*	0.03 (0.10)	0.04 (0.15)	0.02 (0.06)	0.48
	*B. uniformis*	0.003 (0.01)	0.004 (0.01)	0.002 (0.01)	0.24
	*B. ovatus*	0.01 (0.02)	0.004 (0.02)	0.01 (0.03)	0.29

1 Means and standard deviations in parentheses.

2 *p* values from the *t*-test comparing between detectable (A-tetra+) and undetectable (A-tetra−) A-tetrasaccharide groups.

3 *p* value calculated as treating each infant gut microbiota species to be independent for each visit.

4 *Bif. kashiwanohense* species shows significant difference between A-tetra+ and A-tetra− subjects, but when the relative abundance is standardized, the significance in the difference disappears.

**Figure 3:**
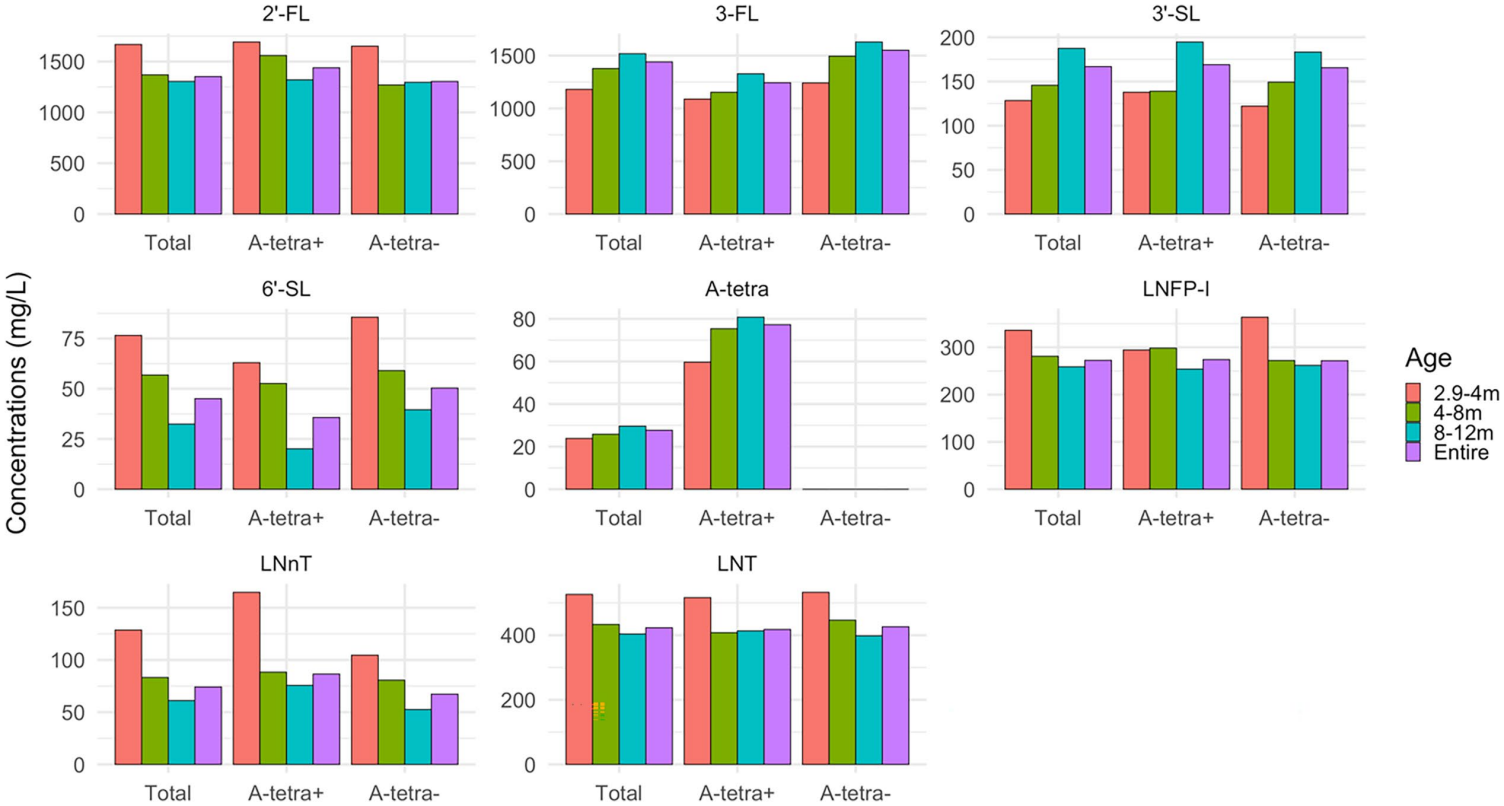
Bar plots showing the apparent temporal variations of HMO concentrations during 2.9–4, 4–8, and 8–12 months, and all HM samples, with and without stratification based on the A-tetra status, respectively. The concentration values are summarized in Supplementary Table 1. 2′-FL, 6′-SL, LNFP-I, LNnT, and LNT decrease while 3-FL, 3′-SL, and A-tetra increase with post-partum age.

Figure 3 shows the HMO concentrations for all HM samples and grouped by post-partum ages: 2.9–4, 4–8, and 8–12 months, both with and without stratification based on the A-tetra status, respectively. The temporal variations of HMO concentrations in relation to post-partum ages are evident. In general, 3-FL, 3′-SL, and A-tetra have an increasing trend and 2′-FL, 6′-SL, LNFP-I, LNnT, and LNT have a decreasing trend by post-partum age. There were no statistically significant differences observed for each HMO between the A-tetra+ and A-tetra− groups across all post-partum age bins. More detailed information of the measured HMO concentrations shown in Figure 3 was provided in the Supplementary Table 1. With the exception of 3-FL (*p* = 0.01, A-tetra+: 1243.26, A-tetra−: 1550.97 mean concentrations), the remaining seven HMOs did not show significant difference between the A-tetra+ and A-tetra− groups.

### 3.2. Specific HMOs, microbial taxa and their interactions associate with cognition

**Figure 4:**
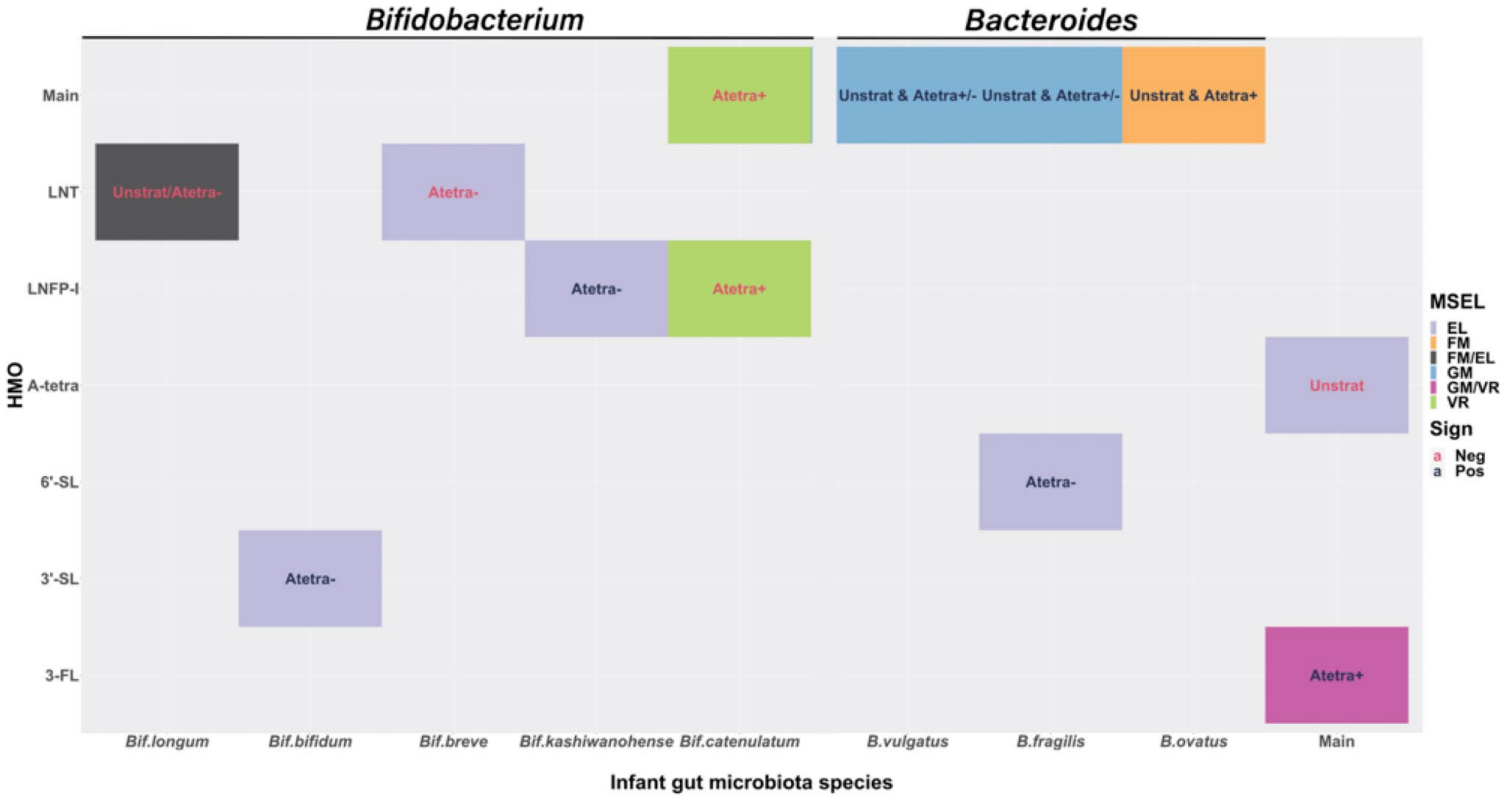
Visual display of the statistically significant fitted results at 0.05 significance level. The filled color indicates different Mullen Scales of Early Learning (MSEL) domains. The text color indicates the sign of the effect size, red for negative and dark blue for positive associations. “Main” in *x*- and *y*-axis are to show the main associations of human milk oligosaccharides (HMOs) and infant gut microbiota relative abundances with cognition. Texts in the figure show from which stratified model the results are: “Unstrat” corresponds to unstratified model, “Atetra+” and “Atetra−” correspond to detectable and undetectable A-tetrasaccharide (A-tetra) stratified models, respectively. “Unstrat & Atetra+/−” corresponds to the results that are shown consistently throughout all three models. “Unstrat & Atetra−” is for the result from unstratified and undetectable A-tetra stratified models and “Unstrat & Atetra+” is for the result from unstratified and detectable A-tetra stratified models. Expressive language (EL); fine motor (FM); Gross motor (GM); and Visual reception (VR).

Figure 4 shows a summary of the main associations between HMOs, *Bifidobacterium* species, *Bacteroides* species, and their respective interactions in association with cognition. To clearly summarize our results, we first provided the associations between HMOs and cognition, as well as microbiota and cognition separately, followed by the results of their interactions in association with cognition below.

#### 3.2.1. Associations between HMOs and cognition

Expressive language had significantly negative association with A-tetra (*p* = 0.026; ES = −2.15) using the unstratified model, suggesting that infants fed with HM containing a high concentration of A-tetra exhibited a lower expressive language score. Additionally, significantly positive associations were observed between 3-FL with visual reception (*p* = 0.041, ES = 6.34) and gross motor (*p* = 0.027, ES = 7.67) in the subjects who received HM with detectable A-tetra (A-tetra+ stratification), indicating that these associations depended on the A-tetra status.

#### 3.2.2. Associations between gut microbiota and cognition

Regardless of the A-tetra status, a higher relative abundance of *B. fragilis* and *B. vulgatus* was significantly associated with a higher gross motor score (*B. fragilis*: unstratified, A-tetra+, and A-tetra−; *p* = 0.017, 0.031, and 0.023; ES = 1.89, 1.75, and 1.69 and *B. vulgatus*: unstratified, A-tetra+, and A-tetra−; *p* = 0.03, 0.039, and 0.027; ES = 1.70, 1.58, and 1.73, respectively). In contrast, fine motor showed significant positive association with *B. ovatus* in both unstratified and A-tetra+ stratified models (*p* = 0.032 and 0.033; ES = 1.94 and 2.15, respectively), indicating that a higher relative abundance of *B. ovatus* was associated with a higher fine motor score. Although both unstratified and A-tetra+ stratified models showed significant associations, the association using the unstratified model was most likely driven by the A-tetra+ group and suggested that the observed association was distinct among infants who were fed with HM with detectable A-tetra.

Finally, a higher visual reception score was significantly associated with a lower *Bif. catenulatum* relative abundance in the subjects who received HM with detectable A-tetra (*p* = 0.012; ES = −2.13), indicating that a higher visual reception score was associated with a lower *Bif. catenulatum* relative abundance in the subjects who received HM with detectable A-tetra.

#### 3.2.3. Associations with cognition through interactions between HMOs and gut microbiota

While the above findings are independent associations between HMOs and species of *Bifidobacterium* and *Bacteroides* with cognition, additional associations through the interactions of the two systems were also observed. Fine motor showed a significantly negative association with the interaction between LNT and *Bif. longum* (ES = −2.12; *p* = 0.037) using the unstratified model. Although not statistically significant, the effect sizes of the main associations of LNT and *Bif. longum* were both negative (−1.07 and − 0.12, respectively). That is, an increase of either LNT concentration, *Bif. longum* relative abundance, or both was associated with lower fine motor scores as seen in the significant interaction effect between LNT and *Bif. longum*.

Visual reception showed a significant negative association with the interaction between *Bif. catenulatum* and LNFP-I (ES = −8.37; *p* = 0.007) in the A-tetra+ stratified model. Again, we examined the effect sizes of the main associations of *Bif. catenulatum* and LNFP-I, which were − 2.13 and 3.41, respectively. That is, a higher relative abundance of *Bif. catenulatum* was associated with lower visual reception scores but the effects would be weakened with an increase of LNFP-I and vice versa.

Finally, expressive language showed significant positive associations with the interaction between 3′-SL and *Bif. bifidum*, 6′-SL and *B. fragilis*, and LNFP-I and *Bif. kashiwanohense* (*p* = 0.01, 0.019, and 0.048; ES = 4.06, 6.95, and 6.31, respectively) and significant negative associations with the interaction between LNT and *Bif. breve* and LNT and *Bif. longum* (*p* = 0.011 and 0.022; ES = −4.19 and − 2.17, respectively) in the A-tetra-stratified model. The main associations of 3′-SL, *Bif. bifidum*, LNFP-I, *Bif. kashiwanohense*, and LNT had negative effect sizes (−0.08, −0.56, −0.91, −2.91, and − 1.68, respectively) while 6′-SL, *B. fragilis*, *Bif. breve*, and *Bif. longum* had positive effect sizes (0.23, 0.61, 0.34, and 0.38, respectively). Thus, associations with expressive language and 3′-SL, *Bif. bifidum*, LNFP-I, *Bif. kashiwanohense*, *Bif. breve*, and *Bif. longum* were diminished due to the significant interactions, while the associations enhanced with 6′-SL and *B. fragilis* in positive direction and with LNT in negative direction.

### 3.3. Adjusting covariates

Significant covariates are summarized in Table 4. For the batch effects, the year 2018 was used as the reference year for the comparisons with years 2019 and 2020. Regardless of the stratification, significant MSEL batch effects were observed for the early learning composite, visual reception, expressive language, and gross motor scores. Fine motor showed a significant batch effect between the years 2018 and 2020 in the unstratified models. Compared to year 2018, with HMOs collected in 2019 or 2020, the above four scores were lower. In general, as the HMOs were collected at later years, the scores decreased. For expressive language, sex of the infants and maternal education also had significant effects in the A-tetra-stratified model. That is, girls compared to boys and infants with college graduated mothers compared to mothers with graduate school level education had higher expressive language scores.

**Table 4:** Statistical results for adjusting covariates using random linear mixed effects model with one random intercept for study subjects.^2^

		Unstratified^3^					A-tetra+^3^				A-tetra−^3^			
		ELC^4^	FM^4^	GM^4^	VR^4^	EL^4^	ELC^4^	GM^4^	VR^4^	EL^4^	ELC^4^	GM^4^	VR^4^	EL^4^
Batch 2019 (reference: 2018)	Estimate	−6.89	−3.34	−5.61	−6.99	−0.84	−7.38	−5.82	−6.80	−1.36	−6.79	−5.35	−8.27	−1.82
	S.E.	2.87	2.41	2.01	1.88	2.00	2.76	1.96	1.85	1.83	2.89	2.00	2.12	1.82
	*t*	−2.40	−1.39	−2.79	−3.72	−0.42	−2.67	−2.96	−3.67	−0.74	−2.35	−2.68	−3.91	−1.00
	*p* value^1^	**0.018** ^5^	0.167	**0.006**	**<0.001**	0.674	**0.009**	**0.004**	**<0.001**	0.459	**0.021**	**0.008**	**<0.001**	0.320
Batch 2020 (reference: 2018)	Estimate	−8.29	−5.85	−5.84	−4.45	−5.46	−9.86	−6.56	−5.62	−5.31	−8.39	−6.12	−4.98	−8.10
	S.E.	3.39	2.84	2.39	2.31	2.37	3.34	2.38	2.29	2.16	3.42	2.37	2.52	2.19
	*t*	−2.44	−2.06	−2.44	−1.93	−2.30	−2.96	−2.76	−2.45	−2.46	−2.46	−2.58	−1.98	−3.70
	*p* value^1^	**0.016**	**0.041**	**0.016**	0.056	**0.023**	**0.004**	**0.007**	**0.015**	**0.015**	**0.016**	**0.011**	0.05	**<0.001**
Sex (Male)	Estimate	−2.51	−0.83	2.36	−0.61	−3.07	−1.77	2.28	0.29	−2.01	−3.17	2.22	0.03	−3.11
	S.E.	2.34	1.83	1.69	1.51	1.65	2.27	1.67	1.49	1.53	2.36	1.67	1.68	1.47
	*t*	−1.08	−0.46	1.39	−0.40	−1.87	−0.78	1.37	0.19	−1.31	−1.35	1.33	0.02	−2.13
	*p* value^1^	0.286	0.650	0.17	0.688	0.066	0.437	0.176	0.848	0.193	0.183	0.188	0.985	**0.037**
Maternal education	Estimate	−2.25	−1.78	−0.33	1.03	−1.41	−2.10	−0.16	0.62	−1.19	−2.22	−0.40	1.16	−2.70
	S.E.	1.69	1.31	1.22	1.09	1.19	1.63	1.20	1.07	1.10	1.70	1.20	1.22	1.10
	*t*	−1.33	−1.36	−0.27	0.95	−1.19	−1.29	−0.13	0.58	−1.08	−1.31	−0.33	0.95	−2.46
	*p* value^1^	0.187	0.177	0.785	0.343	0.240	0.202	0.898	0.565	0.281	0.195	0.743	0.343	**0.016**
Delivery mode (Vaginal)	Estimate	0.48	2.76	0.04	−3.10	−0.51	0.36	0.28	−3.34	−0.54	−0.78	−0.11	−3.08	−2.22
	S.E.	2.86	2.29	2.06	1.78	2.01	2.73	2.01	1.78	1.82	2.93	2.06	2.12	1.85
	*t*	0.17	1.21	0.02	−1.74	−0.25	0.13	0.14	−1.87	−0.30	−0.27	−0.06	−1.46	−1.20
	*p* value^1^	0.868	0.229	0.986	0.084	0.800	0.896	0.176	0.063	0.768	0.792	0.957	0.147	0.233
Site (UNC)	Estimate	2.77	2.65	3.55	1.42	−0.55	3.34	4.08	1.00	0.30	2.56	3.31	1.88	0.37
	S.E.	3.13	2.59	2.25	2.08	2.20	3.21	2.33	2.15	2.08	3.29	2.32	2.36	2.04
	*t*	0.89	1.02	1.58	0.68	−0.25	1.04	1.76	0.47	0.14	0.78	1.43	0.80	0.18
	*p* value^1^	0.379	0.309	0.118	0.496	0.804	0.304	0.083	0.643	0.887	0.44	0.158	0.428	0.855

1 Significant results with 0.05 significance level provided.

2 Results shown from all three models, unstratified, stratified by detectable (A-tetra+), and undetectable (A-tetra−) A-tetrasaccharide models.

3 Unstratified model: the human milk samples used from all the subjects; stratified models: the human milk samples used from only the corresponding subjects, i.e., subjects from the A-tetra+ group for the A-tetra+ stratified model and from the A-tetra− group for the A-tetra− stratified model.

4 Abbreviations for the Mullen Scales of Early Learning scores: ELC for early learning composite score, FM for fine motor, GM for gross motor, VR for visual reception, and EL for expressive language scores.

5 Bold values indicate statistical significance.

## 4. Discussion

Although the conceptual frameworks suggesting a potential association between HMOs, gut microbiota composition, and cognition have been proposed (63), studies thus far have largely focused on the associations between infant cognition and gut microbiota or HMOs, separately. While our findings are consistent with that reported in the literature demonstrating that specific HMOs and *Bifidobacterium* and *Bacteroides* species are independently associated with certain cognitive domains, this study took one step further to provide evidence on the associations between interactions of HMOs and specific microbial taxa and cognitions during the first year of life. Interestingly, most of the HMOs that showed a significant main association with cognition did not show a significant interaction effect except for the interaction between *Bif. catenulatum* and LNFP-I with visual reception. In addition, most of the significant interactions were with expressive language in the A-tetra-stratified model. In general, except for the significant association between *B. fragilis* and 6′-SL with expressive language, the species showing significant interaction with HMOs were *Bifidobacterium* species, while *Bacteroides* species showed significant main associations with motor functions. To the best of our knowledge, our study is the first to evaluate associations between the three biological systems—relative abundance of *Bifidobacterium* and *Bacteroides* species, HMO concentrations, and cognition in typically developing infants during the first year of life. More detailed discussions on the observed associations with specific cognitive abilities are provided below.

### 4.1. Motor skills

Although the potential relations between HMOs and gut microbiota and motor functions during early infancy remain elusive, few reports are available in the literature and results are somewhat inconsistent among these studies. Using gut microbiota coabundance at infancy, Sordillo et al. (64) reported that poor fine motor scores at 3-year old were associated with *Bacteroides*-dominated coabundance group. However, Tamana et al. (65) reported a positive association between motor development at 2 years old and *Bacteroides*-dominant cluster at birth. Finally, by stratifying healthy infants at 18 months old into two groups, above and below median fine motor scores, Acuna et al. (66) reported that probiotic *Bifidobacterium* was more abundant in the above median group. While discrepancies in approaches and ages among study cohorts may have contributed to these inconsistent results, our findings may offer additional insights into the complex associations between HMOs, gut microbiota, and motor functions during infancy. Specifically, our results elucidated that the observed associations differed between gross and fine motor abilities. Gross motor was independently associated with the HMO 3-FL and the microbes *B. fragilis* and *B. vulgatus*. In contrast, no association between HMOs and fine motor was observed. Instead, fine motor was associated with *B. ovatus*, which differs from those with gross motor and through interaction between LNT and *Bif. longum*. To this end, our findings signify the importance to consider potential interactions between HMOs and gut microbiota to more comprehensively analyze the potential relations among HMOs, gut microbiota and motor functions.

It should be noted that although both gross and fine motor assess infants’ motor ability, a potential association between fine motor and language abilities has been reported; fine motor skills at 6–24 months positively predicted the expressive language skill at 30–36 months in autistic children (67, 68). Our results may also support the associations between language and motor ability, as we identified that both expressive language and fine motor skills showed a negative association with the interaction between LNT and *Bif. longum*.

### 4.2. Language skills

Expressive language is the ability to communicate and express thoughts and feelings, while receptive language concerns understanding of information (69). We previously reported that both receptive and expressive language abilities were significantly and positively associated with 3′-SL in infants who received HM containing detectable A-tetra but not in the A-tetra− group (21). In particular, a stronger association between 3′-SL and receptive language was observed in children older than those younger than 1 year-old while no age interaction was observed for expressive language. These previous findings, however, differ from the current study. With the exception of A-tetra, which exhibited a negative association using the unstratified model, all identified associations with language ability in this study were through interactions between microbiota and HMOs, including 3′-SL with expressive language in the A-tetra− group. In addition, no association with receptive language was observed. Although several potential factors may account for the observed discrepancies between our previous and current studies, one of the most plausible reasons is the age differences between the two studies. The age range was 2–25 months old in our previous study while the current study focused on the first 12 months of life. As indicated above, children older than 12 months old exhibited a stronger association between 3′-SL and receptive language than those younger than 12 months old. We further re-analyzed our data using the previous approach but only included subjects in the first year of life, identical to this study, and no significant association was observed (Supplementary material, entitled “Association analyses between infant cognition and HMOs”). Therefore, the difference in age ranges between the two studies might explain why no association with receptive language was observed in the current study. Furthermore, the potential interaction between HMOs and gut microbiota was not accounted for in our previous study. Contrasting to our previous findings, which were specific to A-tetra+ group, inclusion of the interactions between HMOs and microbiota yielded previously unseen associations beyond language ability. These findings underscore the importance of considering the potential interactions between HMOs and gut microbiota in the identification of potential associations between HMOs and cognition. While the underlying mechanisms explaining our findings remain elusive and beyond the scope of the current study, *Bif. breve*, *Bif. longum*, and *Bif. bifidum* showed the highest relative abundances suggesting they have a competitive advantage in the presence of HMOs during breastfeeding. James et al. (70) demonstrated that species from *Bifidobacterium* genus, including *Bif. breve* and *Bif. longum*, but especially, *Bif. breve* are able to metabolize LNT. Notably, LNT is the highest in HM of mothers who cannot express LNFP-I and A-tetra (non-secretor) (47, 48), and Guo et al. (71) showed that *B. fragilis* has an important transglycosylation activity for synthesizing sialylated HMO. The present study findings support these previously published interactions.

Finally, significant associations between the expressive language skills and infant sex and maternal education level in A-tetra− group were observed where girls exhibited a better expressive language ability than boys. This is consistent with the widely accepted intuition that girls are commonly known to have better expressive language skills (72).

### 4.3. Visual reception skill

Mullen Scales of Early Learning visual reception assesses an infant’s ability to understand what different shapes, words, and symbols represent as well as the recognition of objects spatially. In this study, associations were observed between visual reception and HMOs (3-FL), gut microbiota (*Bif. catenulatum*), and their interactions (*Bif. catenulatum* and LNFP-I). Interestingly, *Bif. catenulatum* is not a dominant *Bifidobacterium* species during infancy, hence not much is studied about this species among infants (73). Liu et al. (74) studied the different genetic make-up of *Bif. catenulatum* in infants and adults and found that *Bif. catenulatum* subspecies *kashiwanohense* is generally more dominant among the infants and is equipped with an alpha-L-fucosidase needed to metabolize HMOs. Also, Ojima et al. (75) suggested that *Bif. catenulatum* utilizes fucosyllactose through its fucosyllactose-binding proteins. Our data support this relationship between *Bif. catenulatum* and LNFP-I and showed that visual reception was associated with the interaction between these two. Furthermore, a previous study by Cabrera-Rubio et al. (76) reported that *Bif. catenulatum* was more prevalent among secretor milk samples compared to non-secretors. In our study, we used the level of A-tetra, equally dependent on the secretor positive status, for stratification, as initially used by Cho et al. (21), and observed that the association between *Bif. catenulatum* and LNFP-I for visual reception was specific for the population that received A-tetra+ HM.

While a few studies exist on *Bif. catenulatum*, to the best of our knowledge, no study has previously reported a potential association between visual reception and HMOs although Carlson et al. reported that visual reception was negatively associated with the alpha diversity of gut microbiome at 2 years of life (22). Our study focused on infants during the first year of age and similarly observed a negative association between the visual reception score and the relative abundance of *Bif. catenulatum*.

### 4.4. A-tetra status

Although secretor status has been commonly used to group HM samples in the literatures (77–81), we instead employed A-tetra status in our study for the following two reasons. First, the reported criteria (80, 81) used to determine secretor status led to relatively uneven sample sizes between secretors vs. non-secretors in our cohort, making it difficult to derive statistically meaningful results. Second, this study further expanded our previous findings (21) by considering how the potential interactions between microbiota and HMOs could lead to previously unseen associations with cognition during early infancy (Please see Supplementary material, entitled “Secretor vs. A-tetra statuses” for more detailed discussion). A-tetra status of HM depends both on the secretor positive status and the blood type of A or AB leading to a subgrouping of secretor positive HM (47–49). In our study, we found significant associations differed according to the stratification based on the A-tetra detectability. In particular, most of the interactions were shown in the stratified models. Yet, independent associations of *Bacteroides* species with cognitive scores were less dependent on the A-tetra status. On the other hand, when the associations of the microbiota were dependent on HMOs through interactions, the associations were more significant when HMOs were stratified by A-tetra detectability. This is intuitive in the sense that when stratified, the HMO components would be more homogenous, and thus would have less deviation, allowing more detection of significant terms.

### 4.5. Temporal variations of HMO concentrations with postpartum age

Human milk (HM) oligosaccharide concentrations in HM are known to vary with postpartum age of lactation (82, 83) with some decreases (e.g., 2′-FL and 6′-SL), while others increase (3-FL; Figure 3). These temporal variations underscore the importance of considering when the HM samples were collected in relation to the post-partum age should one be interested in comparing the HMO concentrations with those reported in the literature. Specifically, there is a common misconception that 2′-FL and 6′-SL are always higher than 3-FL and 3′-SL, respectively, because most studies have analyzed samples collected during early infancy, e.g., first 1–2 months of lactation. However, since 2′-FL and 3-FL exhibited an opposite temporal pattern, 2′-FL was higher than 3-FL in 2.9–4 months but became lower in 8–12 months (Figure 3). Likewise, similar findings were observed for 3′-SL and 6′-SL.

Comparing the HMO concentrations in our study to those reported in the literature, Thurl et al. (79) showed that 2′-FL, 3-FL, 3′-SL, and 6′-SL were 3.13, 0.42, 0.27, and 1.22 g/L for secretors and not detected, 1.79, 0.24, and 1.14 g/L for non-secretors for HM samples collected from German women between days 3–90 postpartum, respectively. Since the secretor status was used by Thurl et al., we employed two approaches to determine the secretor status in our cohort and only included HM samples collected between 2.9 and 4 months post-partum for a fair comparison. Specifically, using 2′-FL/3-FL abundance ratio > 6.5827 as the secretor-positive (80), none of our cohort with HM samples collected in 2.9–4 months post-partum met this criterion as secretor positive. In contrast, using 2′-FL < 15 mg/L (81) as non-secretors, nine subjects were secretors and one was non-secretor among the HM samples collected within 2.9–4 months post-partum. The median values of 2′-FL, 3-FL, 3′-SL, and 6′-SL were 1.86, 0.97, 0.12, and 0.076 g/L for secretors and not-detected, 2.43, 0.13, and 0.11 g/L for the non-secretor. It appears that our results are different from that reported by Thurl et al. (79). Several factors may account for the observed discrepancies between ours and those reported by Thurl et al. (79). Specifically, as discussed above, HMO concentrations are anticipated to vary with post-partum age. Since our samples were largely collected beyond the first 3 post-partum months (two samples <90 days post-partum), it is highly likely that our results would be different from that reported by Thurl et al. (79). In fact, our results of 6′-SL were highly consistent with those reported by Austin et al. (49) (76.6 mg/L at 2.9–4 months and 56.8 mg/L at 4–8 months in our study vs. 78 and 39 mg/L for 2–4 and 4–8 months, respectively). Furthermore, several additional publications have reported a wide range of HMO concentrations among different studies, races, geographic locations, and post-partum ages (84–86). Our results are well within the ranges of the reported results. Finally, the age effects were regressed out prior to conducting the association analyses in our study, further minimizing the potential impacts of the temporal variations of HMO concentrations to our conclusions. In summary, although discrepancies of HMO concentrations were observed between ours and those reported by Thurl et al. (79), our results are within the ranges reported by many other studies (49).

### 4.6. Hypothesis on mechanisms of action mediating the HMO and microbiota effects and their interactions

Our findings confirm the hypothesized interaction between HMOs, microbiota, and cognitive functions in infants. Although some of our findings align with existing literature and offer additional insights into the complex triad relation between HMOs, infant gut microbiota, and cognition, this area of research remains largely underexplored. To this end, we offered potential mechanistic hypotheses underlying our findings. In this exercise, we will be obliged to leverage the existing evidence collected in animal models that mainly investigated the HMOs, 2′-FL, 3′-SL, and 6′-SL thanks to their availability for research and/or presence in rodent milk. While potential extrapolation of similar hypothetical mechanisms of action (MoA) to other HMOs beyond these is not excluded, the hypothesis generated will be stronger for these three HMOs. The most obvious MoA via which HMOs can influence subjects is the modulation of microbiota composition and function, which is a subject of intense research in infants (59) as well as in models to study more species-specific growth and activity on specific HMOs (87). HMO boosted microbial metabolites are of particular interest as many of those can go systemic and affect distant sites like the developing brain (42). In this direction, several studies have reported that short-chain fatty acids (SCFA) could be essential. Extensive investigation of the growth of bacteria and their production of SCFA has been reported by Yu et al. (57). In summary, while most *Bifidobacterium* and *Bacteroides* were reported to grow on fucosylated HMOs (2′-FL, 3-FL, and lactodifucotetraose) and generate abundant SCFA, supplementation with sialylated HMOs (3′-SL and 6′-SL) only promoted moderate growth of specific *Bifidobacterium* and *Bacteroides*, which however also generated significant SCFA. Interestingly, SCFA have been associated with neurogenesis (88) as well as potential impact on microglia (89). Beyond SCFA, other metabolites could be mediating the potential impact of HMOs on cognition. Among the candidates are neurotransmitter, such as gamma-aminobutyric acid (GABA) (90) and serotonin (91, 92) or their precursors, such as tryptophan for serotonin (93, 94). Change in gut synthesis of these metabolites can have impacts on brain via various routes, including directly reaching the brain, providing the precursor for brain synthesis of the neurotransmitters, and direct impact on the enteric nervous system or on the vagus nerve (95). Interestingly several probiotics, including *Bifidobacterium*, have been reported to synthesize GABA (90, 96), which would act mostly at the enteric nervous system or via the vagus nerve. Further evidence comes from studies where intervention with probiotics led to changes in behavior or gene expression in the brain. For example, treatment with a lactobacillus strain was shown to impact both central GABA receptor expression as well as emotional behavior via the vagus nerve (97).

Another MoA reported in multiple preclinical studies is the modulation of brain gene expression, which has been studied across different species (42, 98). Fleming et al. used 2′-FL as nutritional intervention, while Hauser et al. used an absence vs. presence of 6′-SL in the early life diet, suggesting that the potential impact on brain gene expression might be different for different HMOs. In these studies, the authors reported alteration of glutamatergic, cholinergic, gamma amino butyric acid-ergic and histone deacetylation pathways in pigs (98) and alteration of neurodevelopment and more specifically myelination pathways in mice (42), respectively. Finally, while some of our findings were consistent with results reported in the literature, such as the modulation of microbiota growth, we also observed unexpected results. Specifically, we observed interactions between 3′-SL and 6′-SL, but not with 2-FL with infant gut microbiota were associated with cognition. Moreover, we also observed interactions with previously unreported HMOs (LNT and LNFP-I) and gut microbiota revealed additional associations with cognition which require future studies to confirm these findings. Going beyond the healthy population, it has been reported that some neurological pathologies, such as autism spectrum disorder (ASD), were associated with gastrointestinal symptoms as well as alteration of the microbiota (99). In preclinical models of autism, administration of bifidobacteria resulted in improved social function (100), possibly via normalization of the immune system. While comprehensive clinical studies in this direction remain lacking, a recent case study utilizing combined oral and enema fecal microbiome transplant for treating late-onset ASD children reported improvement of ASD symptoms in six of nine subjects who were 8 years old or younger (101). These studies suggest that intervention targeting microbiota could be an appealing approach for neurological pathologies, such as ASD. To this end, our results would benefit to be explored further for its potential clinical applications beyond the healthy population.

### 4.7. Limitations

While our results are informative, our study has several limitations. First, the maternal education of our cohort was higher compared to the general population. Second, while the batch effects of the HMOs were included as one of the adjusting covariates, the significant difference between the batches indicated potential limitations on the consistency of the HMOs analyzed. Third, to minimize potential confounding factors from complementary food interacting with gut microbiome, our study only focused on the first year of life since it is a time period when most infants still consumed HM. Fourth, while most of the reported results regarding the potential interactions between gut microbiota and HMOs have largely focused on *Bacteroides* and *Bifidobacterium* genera, including additional gut microbiome genera could have given a more comprehensive understanding of the associations between gut microbiota and HMOs, but would have required a larger sample size. Fifth, it has been suggested that the relative abundances of *Bifidobacterium* and *Bacteroides* in the breastfed infant colon are different depending on the secretor status (102, 103). Future studies could also consider this potential factor. Finally, while it is highly plausible that interactions between HMOs and gut microbiota may also be associated with other developmental outcome, e.g., infant temperament, our study only focused on infant cognition.

## 5. Conclusion

The associations of the relative abundance of the species of *Bacteroides* and *Bifidobacterium* genera, HMO concentrations, and their interactions with infant cognition scores using MSEL were examined. Most of the significant interactions between the gut microbiota relative abundance and HMOs were associated with expressive language ability in A-tetra-HM fed group. Motor scores showed significant positive associations with *Bacteroides* species. Visual reception showed significant associations with *Bif. catenulatum* and HMOs. Our results not only support independent associations of HMOs or gut microbiota with infant cognition, but also showed the importance of considering the interactions between the two systems by unveiling additional associations with cognition during the first year of life. Overall, our study sheds light on the complexity of the interaction between HMOs, infant gut microbiota, and cognition, and the need for further research in this area. Understanding these interactions could have important implications for infant nutrition and development, as well as potential therapeutic interventions for cognitive and neurological disorders later in life.

## Data availability statement

The original contributions presented in the study are publicly available. This data can be found here: https://www.ebi.ac.uk/ena/browser/view/PRJEB63183.

## Ethics statement

The studies involving human participants were reviewed and approved by University of North Carolina at Chapel Hill and University of Minnesota. Written informed consent to participate in this study was provided by the participants for both themselves and their infants.

## Author contributions

BH, JE, and WL designed the research. KB, BH, HH, JE, and WL conducted the research. TL, TS, SC, HZ, and WL analyzed the data or performed the statistical analysis. SC, TL, TS, JH, NS, and WL wrote the manuscript. All authors contributed to the article and approved the submitted version.

## Funding

The sources of support include grants, fellowships, and gifts of materials. This study was supported in part by NIH grants [U01MH110274 (WL and JE); R01MH116527 (TL); R01MH086633 (HZ); and MH104324-03S1 (JE)], and MH015755 (BH), and a grant (WL) from Nestlé Product Technology Center-Nutrition, Société des Produits Nestlé S.A., Switzerland. BH was an iTHRIV Scholar. The iTHRIV Scholars Program was supported in part by the National Center for Advancing Translational Sciences of the National Institutes of Health under Award Numbers UL1TR003015 and KL2TR003016. The funder (Nestlé Product Technology Center-Nutrition, Société des Produits Nestlé S.A., Switzerland) was involved in the study design for collecting HM and stool samples, interpretation of data, writing of this article and the decision for submission.

## Conflict of interest

TS, JH, and NS are employees of Société des Produits Nestlé, SA, Switzerland. This study was supported in part by a grant from Nestlé Product Technology Center-Nutrition, Société des Produits Nestlé S.A., Switzerland. WL was a consultant of and received travel support from Nestlé SA, Switzerland.

The remaining authors declare that the research was conducted in the absence of any commercial or financial relationships that could be construed as a potential conflict of interest.

## Publisher’s note

All claims expressed in this article are solely those of the authors and do not necessarily represent those of their affiliated organizations, or those of the publisher, the editors and the reviewers. Any product that may be evaluated in this article, or claim that may be made by its manufacturer, is not guaranteed or endorsed by the publisher.

## Abbreviations

2′-FL: 2′-Fucosyllactose
3-FL: 3-Fucosyllactose
3′-SL: 3′-Sialyllactose
6′-SL: 6′-Sialyllactose
A-tetra: A-Tetrasaccharide
A-tetra+: Detectable A-tetrasaccharide
A-tetra−: Undetectable A-tetrasaccharide
ES: Effect size
HM: Human milk
HMO: Human milk oligosaccharide
LASSO: Least absolute shrinkage and selection operator
LMEM: Linear mixed effects model
LNT: Lacto-N-tetraose
LNnT: Lacto-N-neotetraose
LNFP-I: Lacto-N-fucopentaose-I.
MoA: Mechanisms of Action
MSEL: Mullen Scales of Early Learning
SCFA: Short-chain fatty acids
