# Supplementary material for: Interactions between Bifidobacterium and Bacteroides and human milk oligosaccharides and their associations with infant cognition
Source: Front Nutr. 2023 Jun 29;10:1216327. doi: 10.3389/fnut.2023.1216327 (PMC10345227; doi:10.3389/fnut.2023.1216327)
Supplement: Supplementary file 1 [file Data_Sheet_1.docx]

Supplementary Material

# Supplementary Figures and Tables

## Supplementary Figures

##
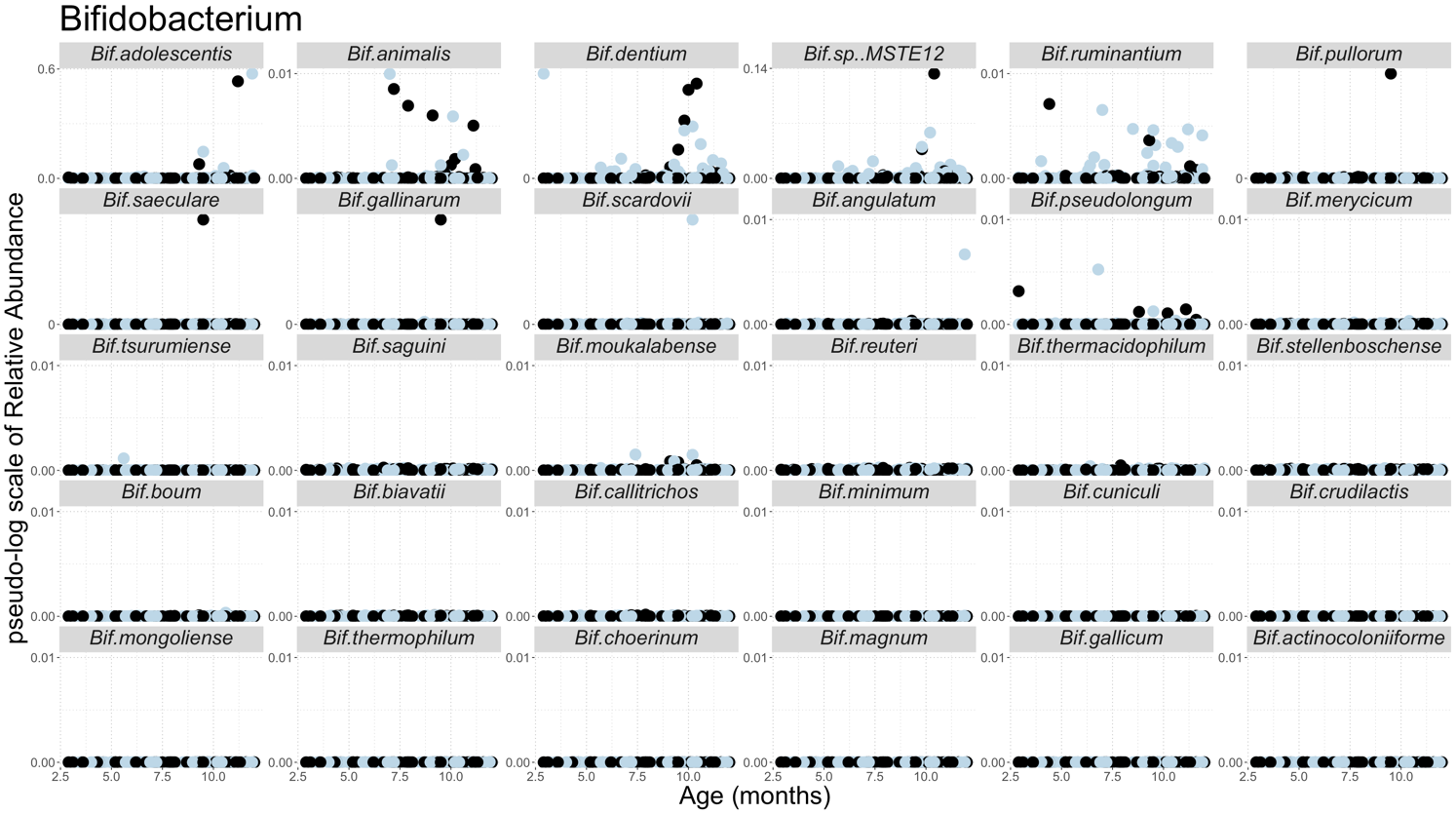

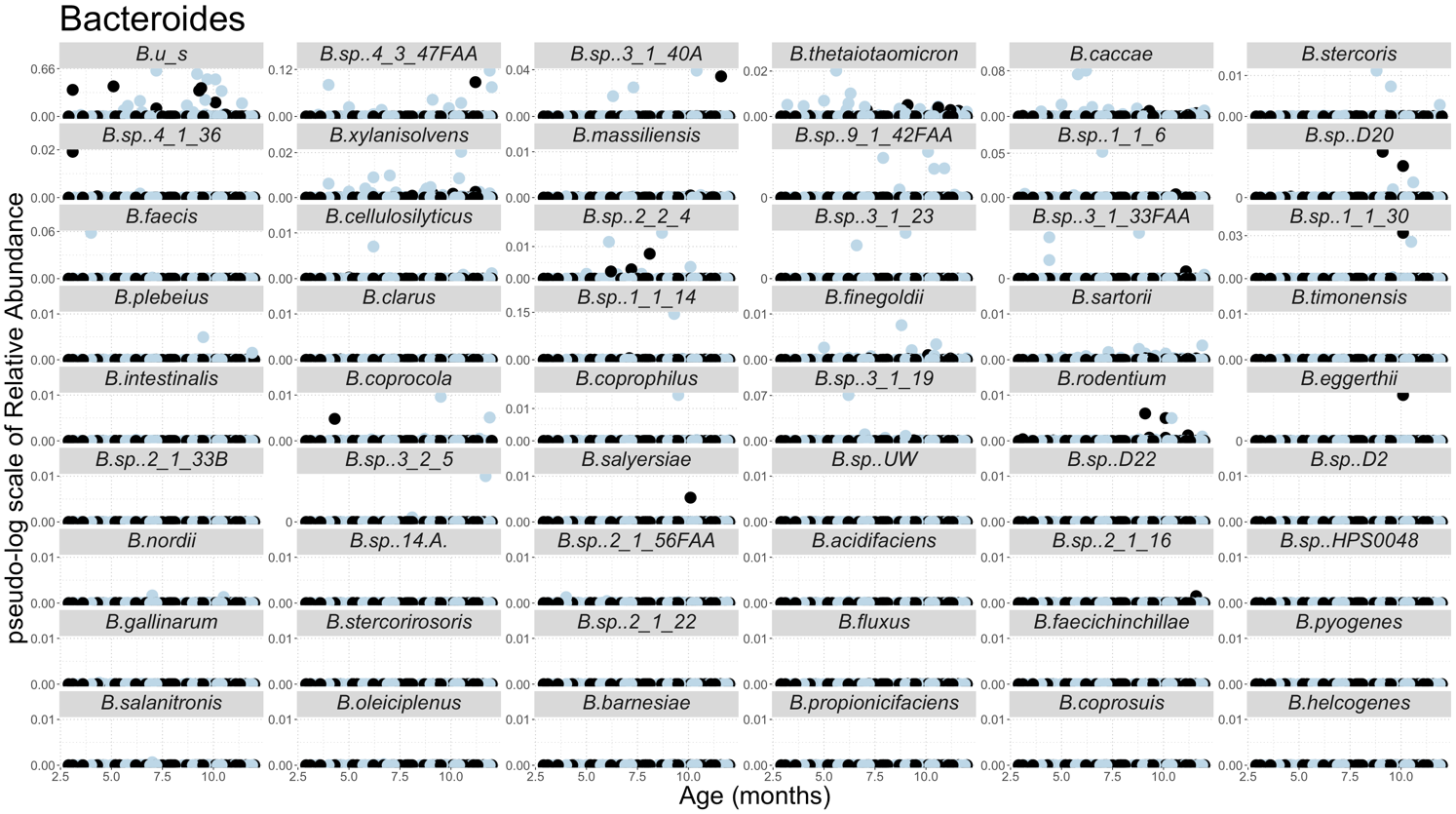


**Supplementary Figure 1.** Scatter plots of the pseudo-log scale of the relative abundance of the remaining of the 37 *Bifidobacterium* and 59 *Bacteroides* species not used in the analyses. The samples in the detectable A-tetrasaccharide (A-tetra) group are colored in black and the ones in the undetectable A-tetra group are colored in light blue. Age in months is indicated in the x-axis.


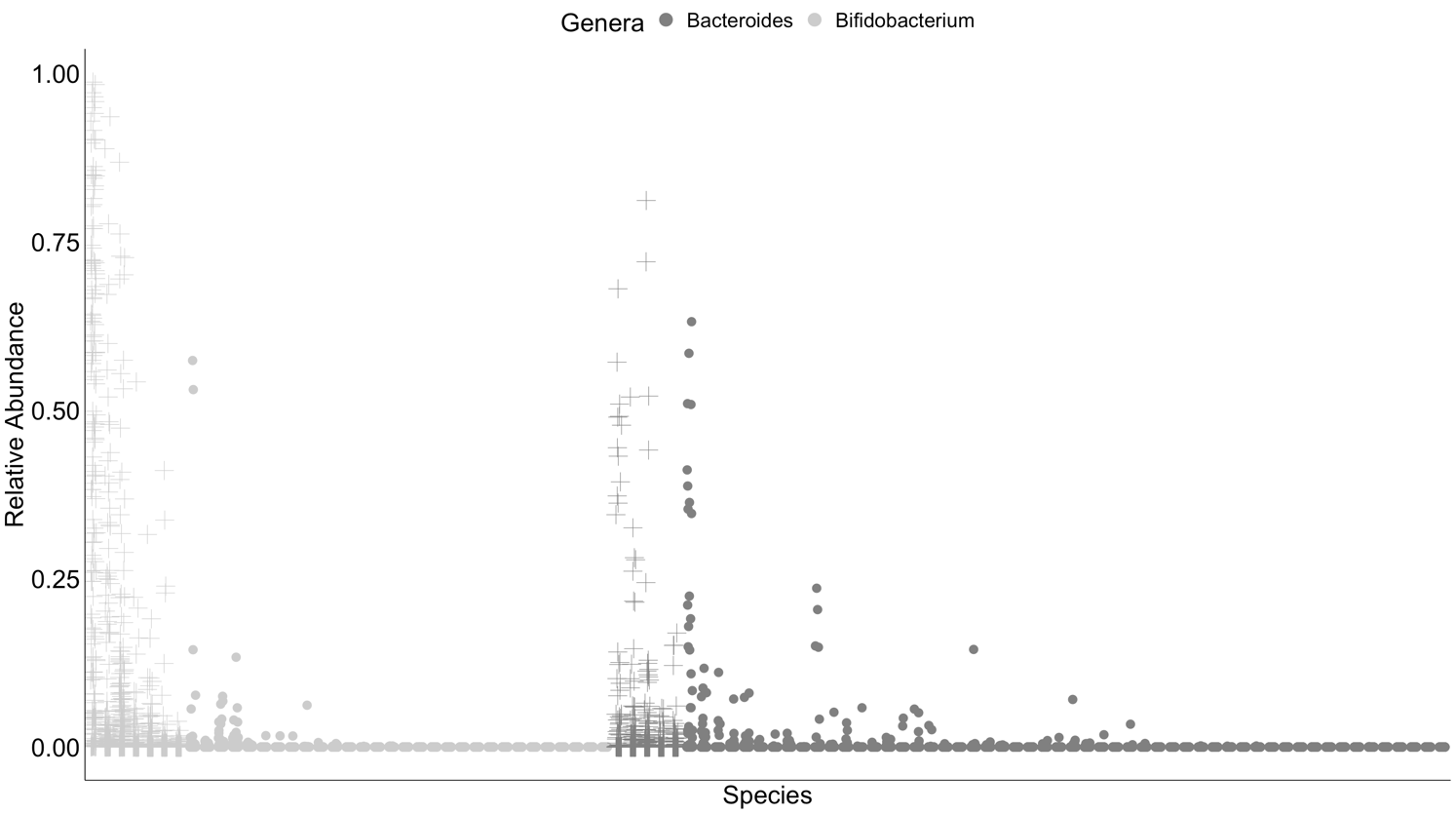


**Supplementary Figure 2.** Strip chart for relative abundances of all the 96 species from *Bifidobacterium* and *Bacteroides* genera. *Bifidobacterium* species in light gray and *Bacteroides* species in dark gray. The twelve species used in the analyses are marked with cross marks and the other species are marked in circles. In general, the twelve species used are the most abundant species.

## Supplementary Table

**Supplementary Table 1. The human milk oligosaccharide concentrations of the participants^1^**

| Human milk  oligosaccharide^5^ | Total (n=170) | | | A-tetra+ (n=61) | | | A-tetra- (n=109) | | | p-value^2,3^ |
| --- | --- | --- | --- | --- | --- | --- | --- | --- | --- | --- |
|  | $\leq$4month  (n=10) | 4-8month  (n=70) | >8month  (n=90) | $\leq$4month  (n=10) | 4-8month  (n=70) | >8month  (n=90) | $\leq$4month  (n=10) | 4-8month  (n=70) | >8month  (n=90) |  |
| 2’-FL | 1352.09 (911.66) | | | 1437.98 (576.15) | | | 1304.02 (1053.40) | | | 0.29 |
|  | 1667.12  (783.30) | 1368.49  (909.13) | 1304.33  (928.29) | 1691.71  (161.65) | 1558.66  (723.64) | 1319.46  (458.38) | 1650.72  (1043.03) | 1269.26  (985.06) | 1295.57  (1117.69) |  |
| 3-FL | 1440.55 (878.66) | | | 1243.26 (542.95) | | | 1550.97 (1005.02) | | | 0.01^4^ |
|  | 1180.37  (651.50) | 1377.72  (860.56) | 1518.34  (912.21) | 1088.90  (465.16) | 1153.05  (559.59) | 1327.58  (539.41) | 1241.35  (789.32) | 1494.93  (966.70) | 1628.77  (1059.42) |  |
| A-tetra | 27.72 (58.59) | | | 77.26 (76.02) | | | 0 (0) | | |  |
|  | 23.87  (35.43) | 25.84  (57.23) | 29.62  (62.01) | 59.67  (30.28) | 75.36  (77.01) | 80.78  (80.20) | 0 (0) | 0 (0) | 0 (0) |  |
| 3’-SL | 166.77 (67.45) | | | 168.99 (77.94) | | | 165.53 (61.17) | | | 0.77 |
|  | 128.40  (30.33) | 145.68  (50.79) | 187.44  (74.83) | 137.78  (23.26) | 138.92  (57.10) | 194.64  (86.70) | 122.15  (34.84) | 149.21  (47.46) | 183.28  (67.50) |  |
| 6’-SL | 45.09 (75.46) | | | 35.72 (31.09) | | | 50.34 (91.09) | | | 0.13 |
|  | 76.57  (29.84) | 56.84  (76.83) | 32.45  (75.93) | 63.00  (25.00) | 52.61  (33.86) | 20.12  (19.27) | 85.62  (31.35) | 59.05  (91.93) | 39.59  (93.86) |  |
| LNT | 423.14 (230.50) | | | 417.87 (210.00) | | | 426.08 (242.10) | | | 0.82 |
|  | 526.07  (190.49) | 433.26  (202.21) | 403.82  (252.72) | 516.29  (188.27) | 407.81  (153.98) | 413.27  (247.01) | 532.59  (209.56) | 446.54  (223.72) | 398.36  (257.98) |  |
| LNnT | 74.18 (69.39) | | | 86.48 (78.22) | | | 67.29 (63.25) | | | 0.10 |
|  | 128.64  (67.67) | 83.24  (70.35) | 61.07  (65.35) | 164.71  (66.57) | 88.29  (65.07) | 75.68  (84.42) | 104.60  (62.04) | 80.60  (73.52) | 52.62  (50.15) |  |
| LNFP-I | 272.53 (293.87) | | | 274.04 (225.68) | | | 271.69 (326.86) | | | 0.96 |
|  | 335.91  (239.54) | 281.07  (291.35) | 258.85  (302.78) | 294.34  (173.23) | 298.30  (239.14) | 253.94  (225.11) | 363.63  (288.06) | 272.08  (317.32) | 261.70  (341.64) |  |

^1^ means and standard deviations in parentheses.

^2^ P-values from the t-test comparing between detectable (A-tetra+) and undetectable (A-tetra-) A-tetrasaccharide groups.

^3^ p-value calculated as treating each concentration to be independent for each visit.

^4^ 3-fucosyllactose shows significant difference between A-tetra+ and A-tetra- subjects.

^5^ Abbreviations for the eight measures are as follow: 2’-FL for 2’-fucosyllactose, 3-FL for 3-fucosyllactose, A-tetra for A-tetrasaccharide, 3’-SL for 3’-sialyllactose, 6’-SL for 6’-sialyllactose, LNT for lacto-N-tetraose, LNnT for lacto-N-neotetraose, and LNFP-I for lacto-N-fucopentaose-I.

**Supplementary Table 2. Comparison between A-tetra and secretor statuses using the criterion from Totten et al.**

|  | A-tetra + | A-tetra - |
| --- | --- | --- |
| Secretor | 60 | 103 |
| Non-secretor | 1 | 6 |

**Supplementary Table 3. Comparison between A-tetra and secretor statuses using the criterion from Wang et al.**

|  | A-tetra + | A-tetra - |
| --- | --- | --- |
| Secretor | 61 | 87 |
| Non-secretor | 0 | 22 |

# Secretor vs A-tetra statuses

Secretor status has been commonly used to group HM samples in various studies, we employed A-tetra status instead in this study. Therefore, we compared the potential differences between these two grouping approaches. Specifically, we employed two different criteria for determining the secretor status in our cohort. Totten et al. [1] using HM samples collected during the first 6 post-partum months in Gambian women and 2’-FL/3-FL abundance ratio > 6.5827 was used as secretor-positive. Supplementary Table 2 shows the comparison between A-tetra and secretor statuses.

In contrast, Wang et al. [2] collected HM samples from Chinese mothers at 1-5 days, 8-14 days, 4 weeks, and 6 months postpartum and 2’-FL<15mg/L was used as the indicator for secretor-negative. Thus, except for the 6 months samples, the samples collected in our study were later in post-partum ages when compared to those collected by Wang et al. [2]. Supplementary Table 3 shows the comparison between A-tetra and secretor statuses using the criterion used by Wang et al. [2].

Note the relatively uneven sample sizes between secretors vs non-secretors, particularly when the 2’-FL/3-FL abundance ratio was employed. In contrast, the ratio between A-tetra+ and A-tetra- samples was about 1:3, which is consistent with our previously reported results [3]. Furthermore, as critically discussed in Cho et al. [3], the A-tetra detectability can also be seen as a result from different maternal characteristics and/or genetic polymorphisms. Not much is studied regarding the potential biological traits leading to women secreting detectable vs undetectable A-tetra. Here, two potential possibilities are offered. First, Sabharwal et al. showed that A-tetra was associated with Lewis b-active LNDFH-I, where only the mothers with blood group A or AB had HM with detectable A-tetra [4]. Second, patients whose urine was accumulated with A-tetra tended to show glycosylation disorder since the deficiency of glucosidase I resulted in excess accumulation of tetrasaccharide in urine [5]. Therefore, we could conceive that glucosidases might play a role in distinguishing between women who secretes detectable vs undetectable A-tetra. Finally, to further extend our previous findings [3], A-tetra, instead of secretor status was employed for grouping HM samples in our study. Indeed, our results demonstrate that not only the main associations but also the interactions between HMOs and gut microbiota in relation to cognitive outcomes differ between A-tetra+ and A-tetra- groups.

# Association analyses between infant cognition and HMOs

When the association analyses are done for the infants in their first year of age with no microbiome species included in the analyses, no significant association is observed between receptive language and 3’-sialyllactose. This result is anticipated as from Cho et al. the significance was observed among the older than one year old infants rather than the younger subjects. Even without microbiome species included, similar results with the main results were observed. From the main analyses, from detectable A-tetra stratified model, significant positive associations with 3-fucosyllactose (3-FL) and visual reception and gross motor were observed. Without microbiome covariates included, the same Mullen score domains also showed positive associations with 3-FL and the significances remained even after the multiple comparison for the p-values (effect sizes 9.553 and 8.746 and adjusted p-values of 0.023 for both visual reception and gross motor, respectively). From the analyses without the microbiome species, from the detectable A-tetra stratified model, gross motor also showed significant negative association with lacto-N-tetraose with an adjusted p-value of 0.045 and an effect size of -8.046. However, this association was not observed from the main analyses. Since the significance is not that strong, the signal might have been obscured by the inclusion of the microbiome species from the main analyses.

1. Totten, S.M., et al., *Comprehensive profiles of human milk oligosaccharides yield highly sensitive and specific markers for determining secretor status in lactating mothers.* J Proteome Res, 2012. **11**(12): p. 6124-33.

2. Wang, M., et al., *Neutral Human Milk Oligosaccharides Are Associated with Multiple Fixed and Modifiable Maternal and Infant Characteristics.* Nutrients, 2020. **12**(3): p. 826.

3. Cho, S., et al., *Human milk 3’-Sialyllactose is positively associated with language development during infancy.* The American Journal of Clinical Nutrition, 2021. **114**(2): p. 588-597.

4. Sabharwal, H., S. Sjöblad, and A. Lundblad, *Affinity Chromatographic Identification and Quantitation of Blood Group A-Active Oligosaccharides in Human Milk and Feces of Breast-Fed Infants.* Journal of Pediatric Gastroenterology and Nutrition, 1991. **12**(4).

5. De Praeter, C.M., et al., *A Novel Disorder Caused by Defective Biosynthesis of N-Linked Oligosaccharides Due to Glucosidase I Deficiency.* The American Journal of Human Genetics, 2000. **66**(6): p. 1744-1756.
